# Role of Calprotectin as a Modulator of the IL27-Mediated Proinflammatory Effect on Endothelial Cells

**DOI:** 10.1155/2015/737310

**Published:** 2015-11-17

**Authors:** Susann A. Dorosz, Aurélien Ginolhac, Thilo Kähne, Michael Naumann, Thomas Sauter, Alexandre Salsmann, Jean-Luc Bueb

**Affiliations:** ^1^Life Sciences Research Unit, University of Luxembourg, 162a Avenue de la Faïencerie, 1511 Luxembourg City, Luxembourg; ^2^Institute for Experimental Internal Medicine, Otto-von-Guericke University Magdeburg, Leipziger Straße 44, 39120 Magdeburg, Germany

## Abstract

An underlying endothelial dysfunction plays a fundamental role in the pathogenesis of cardiovascular events and is the central feature of atherosclerosis. The protein-based communication between leukocytes and inflamed endothelial cells leading to diapedesis has been largely investigated and several key players such as IL6, TNF*α*, or the damage associated molecular pattern molecule (DAMP) calprotectin are now well identified. However, regarding cytokine IL27, the controversial current knowledge about its inflammatory role and the involved regulatory elements requires clarification. Therefore, we examined the inflammatory impact of IL27 on primary endothelial cells and the potentially modulatory effect of calprotectin on both transcriptome and proteome levels. A qPCR-based screening demonstrated high IL27-mediated gene expression of* IL7*,* IL15*,* CXCL10*, and* CXCL11*. Calprotectin time-dependent downregulatory effects were observed on IL27-induced* IL15* and* CXCL10* gene expression. A mass spectrometry-based approach of IL27 ± calprotectin cell stimulation enlightened a calprotectin modulatory role in the expression of 28 proteins, mostly involved in the mechanism of leukocyte transmigration. Furthermore, we showed evidence for STAT1 involvement in this process. Our findings provide new evidence about the IL27-dependent proinflammatory signaling which may be under the control of calprotectin and highlight the need for further investigations on molecules which might have antiatherosclerotic functions.

## 1. Introduction 

Atherosclerosis is characterized by the narrowing of arteries caused by plaque formation and is a prominent representative of cardiovascular diseases [1, 2]. Interestingly, development of atherosclerosis occurs often prior to other cardiovascular events such as strokes or heart attacks. Under healthy conditions, the vascular system contains an endothelium composed of a monolayer of cells and forms a barrier between the circulating blood and the vessel wall [3]. The autocrine, paracrine, and endocrine mechanisms of the vascular endothelium can exert modulatory effects like the capacity to regulate cell activation as well as proliferation influencing the growth and metabolism of the surrounding tissue [4]. Furthermore, it has a key role being a gatekeeper by regulating leukocyte trafficking between the blood and the underlying tissue [5]. Alterations of endothelial functions can be caused by several risk factors including smoking, hypercholesterolemia, hyperglycemia, genetic factors, hypertension, ageing, or inflammation [1, 2, 6–8]. While a normal quiescent endothelium induces almost no expression of proinflammatory molecules [6], the recognition of endo- and exogenous danger signals by endothelial cells (ECs) can lead to inflammatory responses through the expression of adhesion molecules, secretion of inflammatory proteins, and morphological changes of ECs [9, 10].

A key event in the early vascular inflammation process is the recruitment and adhesion of leukocytes prior to transendothelial migration into inflammatory sites, a mechanism which involves cytokines, hormones, pathogen associated molecular pattern molecules (PAMPs), and damage associated molecular pattern molecules (DAMPs) [11, 12]. The role of cytokines and DAMPs, especially their atherogenic activities and their involvement in the acceleration of vascular diseases, is already well documented [13–18]. Regarding the IL6-family member IL27 cytokine, composed of the subunits p28 and Epstein-Barr-virus-induced gene 3 (EBI3), its participation in immune responses and diseases is more and more accepted [19]. Contradictory observations regarding its pro- or anti-inflammatory role are however reported [20, 21]. It has been shown* in vivo* that IL27 reduced inflammation by suppressing excessive Th1 immune responses during infection, and* in vitro* in several T cell subtypes it has been shown that IL27 induced the production of the anti-inflammatory IL10 [22, 23]. More recently, the anti-inflammatory role of IL27 as an upstream activator of the STAT3 pathway was also established [24]. In the context of atherosclerosis, Hirase and coworkers demonstrated that mice with IL27 receptor deficiencies develop atherosclerotic lesions [25].

On the other side, other data rather strive for proinflammatory activity of IL27, as described by Guzzo and collaborators in primary monocytes [26] or by Nam and coworkers who demonstrated that IL27 is secreted from pre- and normal adipocytes under inflammatory conditions [27]. Moreover, in terms of atherosclerosis development, IL27 is known to induce in HUVECs the upregulation of the chemokines CXCL9 and CXCL10, implicated in the transendothelial cell migration [19, 28]. In a human study, higher serum levels of IL27 were detected in patients suffering from coronary artery diseases (CAD) such as myocardial infarction and stable and unstable angina pectoris [29]. A pathway analysis of primary tissue from different coronary atherosclerotic lesion demonstrated an upregulation of IL27 in the early developed lesions of atherosclerotic material, which emphasizes an important role of IL27 in the development of atherosclerosis [30]. Altogether these contradictory data about the exact role of IL27 in inflammation suggest the existence of cytokine-specific regulation processes occurring during cell to cell communication.

Calprotectin, a S100A8/S100A9 heterodimer member of the S100 protein family, is also known to be involved in acute and chronic inflammation [31, 32]. Most of the publications propose a proinflammatory function for calprotectin, and regarding the development and progression of atherosclerosis, both* in vitro* and* in vivo* studies suggest a proatherogenic role for calprotectin [33–36]. On the contrary, it has been reported that administration of calprotectin induces immunosuppressive functions in rat animal models [37, 38], indicating that, similar to IL27, calprotectin may have opposite regulatory functions.

In this study, we enlightened the involvement of IL27 and calprotectin in the regulation of the inflammatory state of the endothelium in terms of pro- and antiatherogenic functions. Moreover, with the aim of identifying potential synergistic, additive, or antagonistic effects from other mediators, we analysed the role of calprotectin in IL27-mediated transcriptome and proteome regulation.

## 2. Materials and Methods

### 2.1. HUVEC Isolation, Purification, and Activation

After informed consent of parturients (Comité National D'Ethique de Recherche Luxembourg 2013/01v1.0), primary Human Umbilical Vein Endothelial cells (HUVECs) were isolated with 1 mg/mL collagenase NB4 (SERVA) from fresh umbilical cord veins from planned C-sections (protocol adapted from [39]). The HUVEC cell cultures were grown on 0.2% gelatine-coated tissue flasks and with complete M199 (SIGMA) supplemented with EGM2 SingleQuots (LONZA, Verviers, Belgium) and 2 mM L-glutamine (SIGMA) at 37°C in humid atmosphere with 5% CO_2_. Purity of HUVEC cell cultures was assessed by flow cytometry; the following antibodies were used: mouse anti-human CD31-PE, mouse anti-human CD144-A647, and mouse anti-human CD146-PerCP-Cy5.5 antibodies (all from BD Sciences, Erembodegem, Belgium). Passage numbers 2–4 were used for the activation of HUVECs. Stimulation experiments with HUVECs were prepared with a cell density of 2.5 × 10^5^ cells/mL in 6-well plates (Greiner, Vilvoorde, Belgium) with 1 mL/well complete M199 media for 24 h, and with a subsequent depletion phase of 12 h with EGM2 SingleQuots-depleted M199 media supplemented with 2% FBS (LONZA), gentamycin (LONZA), and 2 mM L-glutamine (SIGMA, MA). To determine the optimal stimulation concentrations, HUVECs were incubated for 12 h with ranges of 10, 30, and 100 ng/mL IL27 (R&D Systems, Abingdon, UK) and 1, 5, and 10 *μ*g/mL calprotectin (Hycult, Uden, Netherlands) (3 biological replicates). In the costimulation assays, for transcriptome analysis, HUVECs were stimulated with IL27 (30 ng/mL) ± calprotectin (1 *μ*g/mL) for 3, 6, 12, and 24 h (6 biological replicates) and for intracellular proteome analysis a time course of 6, 12, and 24 h was performed (9 biological replicates). Furthermore, HUVECs were stimulated with TNF*α* (2 ng/mL) (Peprotech, NJ)** ±** calprotectin (1 *μ*g/mL) for 3, 6, 12, and 24 h (3 biological replicates). Stimulations were stopped by washing the adherent HUVECs with 1 mL of ice cold sterile PBS and the 6-well plates were directly frozen at −80°C. Cell viability was assessed by LDH release using cytox96 nonradioactivity cytotoxicity assay (Promega, Leiden, Netherlands).

### 2.2. RNA Extraction and RT-qPCR

RNA extraction from HUVECs was performed according to the instructions and solutions of the ReliaPrep RNA Cell Miniprep System (Promega). 250 *μ*L of the lysis buffer was added to each well and cells were scratched off. Afterwards, purified RNA was eluted with 30 *μ*L nuclease-free water. RNA yield and purity were determined by spectrophotometer NANOdrop 2000 (Thermo Scientific, CA). For reverse transcription, 0.2 *μ*g random primer (Promega) was added to 1 *μ*g RNA and incubated for 5 min at 70°C, followed by a subsequent addition of 5x reaction buffer, 20 U (final) RNAsin Ribonuclease inhibitor (Promega), 0.5 mM (final) dNTP (Promega), and 160 U (final) GoScript reverse transcriptase (Promega) with incubations of 5 min at 25°C, 60 min at 42°C, and 10 min at 70°C.

For determination of optimal stimulation concentration, a 96-multigene array TaqMan Human Immune panel (cat. 4370573) was used. The cDNA (437.5 ng) was mixed with TaqMan Universal Master mix II (Applied Biosystems, CA) and added per well. Parameters for qPCR were set as follows: 2 min at 50°C, 10 min at 95°C, and 40 repetitions of 15 s at 95°C and 60 s at 60°C (QuantStudio 12K Flex v1.1, Applied Biosystems). Data analysis was performed by Expression Suite v1.1 software. For time course analysis, the following TaqMan primers were used: IL7 Hs00174202_m1, IL15 Hs00174106_m1, CXCL10 Hs00171042_m1, CXCL11 Hs00171138_m1, and the HKGs ACTB Hs99999903_m1, GAPDH Hs99999905, and GUSB Hs99999908_m1. In total, 25 ng of cDNA was mixed with TaqMan Universal Master mix II (Applied Biosystems) and added per well of a MicroAmpFast 96-well plate (Applied Biosystems). Parameters for qPCR were set as follows: 10 min at 93°C and 40 repetitions of 15 s at 95°C and 60 s at 60°C (QuantStudio 12K Flex v1.1, Applied Biosystems).

Quantitative analysis was performed by the method of Willems and collaborators [40]. Internal normalization was performed to ACTB, GAPDH, and GUSB housekeeping genes (HKGs).

### 2.3. Proteomics

#### 2.3.1. Sample Preparation

For cells lysis, 50 *μ*L 8 M urea was added directly to each well and cells were scratched off, transferred, and subsequently sonicated for 1 h at 4°C. Afterwards 200 *μ*L of 0.8% RapiGest (Waters) within 50 mM NH_4_HCO_3_ and 2 mM DTT were added and incubated for 1 h at room temperature (RT), followed by addition of 10 mM MMTS (Pierce, Erembodegem, Belgium) and incubation of 1 h at RT in the dark. Digestion of proteins in peptides was achieved by addition of 1 *μ*g Trypsin Gold (Promega) for 24 h at RT and stopped by addition of 1% TFA for 1 h at RT. Afterwards samples were salted out by using 3 M Empore cartridges (3 M Bioanalytical, MS). Samples were loaded on cartridges, eluted with 200 *μ*L of 70% acetonitrile (ACN) within 0.1% TFA, and further dried in vacuum centrifuge (Speedvac, Thermo Scientific). Dried samples were resuspended with 20 *μ*L of 0.1% TFA. Samples were loaded on Zip Tips C18 (ZTC18S960, Millipore, Molsheim, France) and eluted with 20 *μ*L 70% ACN within 0.1% TFA and dried in vacuum centrifuge (Speedvac, Thermo Scientific). Before measurement, samples were resuspended in 10 *μ*L of 2% ACN within 0.1% TFA.

#### 2.3.2. LC-MS/MS

Peptide analysis was performed by LC-MS/MS on an EASY-nLC Ultra HPLC (Thermo Scientific) coupled to a hybrid dual-pressure linear ion trap/orbitrap mass spectrometer (LTQ Orbitrap Velos Pro, Thermo Scientific). Dissolved peptide samples were separated on a 75 *μ*m (inner diameter), 25 cm, PepMap C18-column (Dionex-Thermo Sciences). A solution gradient ranging from 2% to 40% ACN within 0.1% formic acid at a constant flow rate of 300 nL/min for 200 min enabled the preseparation of peptides. Eluting peptides were ionized in a nanospray interface. Regarding the MS/MS settings, collision-induced dissociation (CID) was applied for the 15 most abundant ions detected in the full MS scan. Essential MS settings were as follows: full MS (FTMS; resolution 60000; *m*/*z* range 400–2000) and MS/MS (linear trap; minimum signal threshold 500; isolation width 2 Da; dynamic exclusion time setting 30 s; singly charged ions were excluded from selection; normalized collision energy was set to 35% and activation time to 10 ms).

#### 2.3.3. Label-Free Quantification

Progenesis QI for proteomics (Waters, Nonlinear Dynamics, MA) was used for label-free quantification of LC-MS derived data. First, alignment of the two-dimensional ion intensity map representing the retention time and mass to charge ratio of peptides was performed, followed by quantification of signals and finally peptide and protein identification by database search. In order to rely on proteins that were confidently identified, a cut-off for at least two unique peptides per protein was applied.

#### 2.3.4. Data Normalization

The progenesis QI for proteomics quantified proteins was still skewed toward high abundances. Thus, abundances were further processed by using *R* (v3.2.1, [41]) for data normalization using the *R* bioconductor package variance stabilization and normalization (VSN [v3.36.0], [42]) and the function* vsn2.*

In order to assess the variability between the biological replicates, calculation of the peak sum per replicate was performed; that is, a ratio between each protein abundance to the sum of all protein abundances of the considered replicate was calculated. Afterwards for each time point, the peak sum medians per replicates and a peak sum median of all replicates were calculated. Application of a cut-off standard deviation (sd) of 1.25 allowed an elimination of outlier replicates. In detail, 2 replicates for the 6 h time point, 6 replicates for the 12 h time point, and 7 replicates for the 24 h time point were removed. Additionally, two biological replicates, control replicates 1 and 2 for *t* = 24 h, despite fulfilling the cut-off of sd of 1.25 showed a shifted distribution and were subsequently removed (Figures S1–S3 in Supplementary Material available online at http://dx.doi.org/10.1155/2015/737310).

#### 2.3.5. Differential Protein Expression Calculation

Retained replicates were implemented to the Linear Models for Microarray Data (*limma*) bioconductor *R* package [v3.24.10], for determination of differential expression [43]. This package was already successfully applied to proteomics [42, 44, 45].* limma* uses an empirical Bayes *t*-test that takes into account the global variance to avoid the noise of local variance for small samples.

Correction for multiple testing was performed by computing the false discovery rate (termed as *q*-values) using the *p* values provided by limma and calculated using the *R* bioconductor package *q*-value [v2.0.0] [46]. Lastly, logarithmic base 2 FC (LogFC) values were converted back to original log2FC using the function sinh. Plots were performed using the* ggplot2 R* package [v1.0.1] [47]. Significance threshold of the *q*-values is determined by plotting *q*-values over *p* values and identification of intersection (Figure S4) [45]. Statistically significant *q*-values were indicated with ^*∗*^ *q* < 0.15, ^*∗∗*^ *q* < 0.10, and ^*∗∗∗*^ *q* < 0.01. Of note, all statistical analyses were performed on a mac OSX architecture (x86_64-apple-darwin14.3.0 (64-bit)).

### 2.4. Western Blot

HUVEC samples were scratched off with a Triton-x lysis buffer. A 10% tris-glycine polyacrylamide gel was prepared and approximately 20 *μ*g protein was loaded (mixed with 5x Laemmli buffer). 3 *μ*L of PageRuler Plus Prestained protein Ladder (Thermo Scientific) was transferred at least to one well, and samples of interest were transferred to free wells of the gel. Furthermore, a 0.2 *μ*m PVDF membrane was used. Antibodies were from BD Biosciences: pSTAT1 pY701 (612233), STAT1 (610116), and STAT3 (610190); cell signaling: pSTAT3 pY705 (9145S); Pierce Thermo: tubulin (PA1-38814). Secondary antibodies were anti-goat IRdye800CW (ODYSSEY, 926-32214) and anti-mouse Alexa Fluor 680 (Invitrogen, A10038). Membranes were detected by fluorescent labelled target proteins, a photo sensor of the LI-COR Odyssey Infra-Red Imaging System (LI-COR, NE). For densitometry analysis, pSTAT1 (*n* = 3) and pSTAT3 (*n* = 3) were normalized to tubulin.

### 2.5. Pathway Analysis

Data were analysed through the use of QIAGEN's Ingenuity Pathway Analysis (IPA, QIAGEN Redwood City; http://www.ingenuity.com/). Normalized transcriptome data of different concentrations per stimulus and proteomic data of 6, 12, and 24 h were merged, respectively. Core analyses were performed with the following settings: *p* value < 0.05 (transcriptome data) or *q*-value < 0.15 (proteome data); reference set was the Ingenuity Knowledge Base (Genes + Endogenous Chemicals), where only experimental observations, direct and indirect relationships, and the Fisher exact *t*-test were chosen; the knowledge bases from all species and all cell types were included. Regarding transcriptome data, a comparative analysis of IL27- and calprotectin-regulated genes was performed and the top 20 biological functions were chosen based on scoring the *z*-scores. Afterwards, a hierarchical clustering was performed on the top 20 biological functions based on Euclidean distance using the function heatmap.2 from the *R* package gplots.

### 2.6. Statistical Analysis

The statistical analysis was performed using the software GraphPad Prism 5 (GraphPad Software, La Jolla, CA, USA). For more than 5 biological replicates and unequal variances, an unpaired *t*-test with Welch's correction and, for less than 5 biological replicates, an unpaired *t*-test were applied. Statistically significant *p* values were indicated with ^*∗*^ *p* < 0.05, ^*∗∗*^ *p* < 0.01, and ^*∗∗∗*^ *p* < 0.001.

## 3. Results

### 3.1. IL27 and Calprotectin-Dependent Regulation of the Endothelial Cell Gene Expression

**Figure 1:**
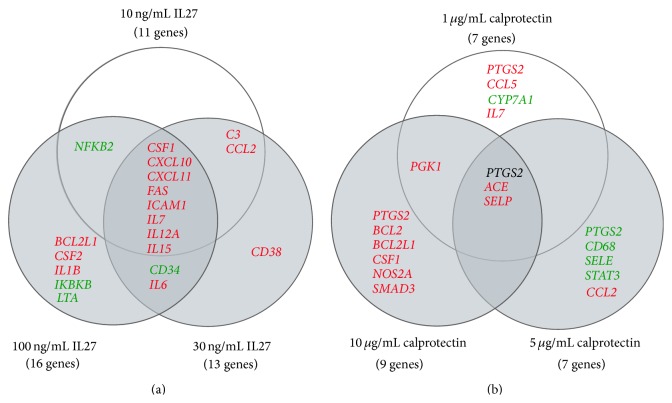
Venn diagrams on IL27- and calprotectin-stimulated HUVECs including significant regulated genes with *p* value *p* < 0.05: red: upregulated, green: downregulated, and black: up- and downregulation different in compared concentrations. (a) Venn diagram: HUVECs stimulated with IL27 (10, 30, and 100 ng/mL). (b) Venn diagram: HUVECs stimulated with calprotectin (1, 5, and 10 *μ*g/mL). ACE, angiotensin-converting enzyme; BCL2, B-cell lymphoma 2; BCL2L1, BCL2-like 1; C3, complement component 3; CD, cluster of differentiation; CSF, colony stimulating factor; CCL2/MCP-1, monocyte chemotactic protein-1; CCL5/RANTES, regulated on activation, normal T cell expressed and secreted; CXCL10, interferon gamma-induced protein 10; CXCL11, interferon-inducible T cell alpha chemoattractant; CYP7A1, cholesterol 7 alpha-hydroxylase; FAS, Fas cell surface death receptor; ICAM1, intercellular adhesion molecule 1; IKBKB, inhibitor of kappa light polypeptide gene enhancer in B-cells, kinase beta; IL, interleukin; LTA, lymphotoxin-*α*; NFKB2, nuclear factor-kappa-B p100 subunit; NOS2A, inducible nitric oxide synthase; PGK1, phosphoglycerate kinase 1; PTGS2, prostaglandin G/H synthase and cyclooxygenase; SELE, selectin E; SELP, selectin P; SMAD3, mothers against decapentaplegic homolog 3; STAT, signal transducers and activators of transcription.

To examine the effect of IL27 and calprotectin on endothelial cell gene expression, a multiplex gene array analysis of 96 genes including cytokines, growth factors, and other immune response genes was performed. In order to optimize the stimulation concentrations for IL27 and calprotectin, 3 different concentrations for IL27 (10, 30, and 100 ng/mL) and for calprotectin (1, 5, and 10 *μ*g/mL) were used, according to published data [19, 33, 48]. The relationship of significant differentially expressed genes following stimulation with IL27 is shown in Figure 1(a). Nineteen unique significantly expressed genes were found among which 15 were upregulated and 4 were downregulated. Interestingly, the highest upregulations were observed for* IL7*,* IL15*,* CXCL10*, and* CXCL11* (Table S1). Regarding calprotectin cell stimulation, 15 unique significantly expressed genes were found, among which 11 genes were upregulated and 4 genes were downregulated, with the gene* PTGS2* being upregulated after 1 and 10 *μ*g/mL calprotectin treatment and downregulated after 5 *μ*g/mL calprotectin stimulation. The highest differential gene expression was observed for IL7 and CCL5 (Table S1).

**Figure 2:**
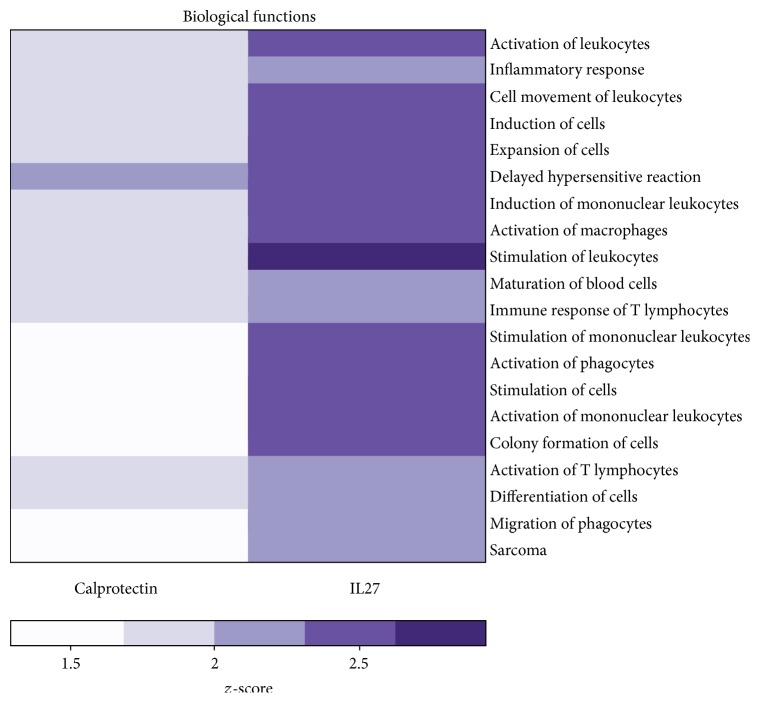
Top 20 biological functions from a comparative gene enrichment analysis of IL27 and calprotectin-mediated gene expression. The biological function gene enrichment analysis was carried out by IPA and represents the *z*-score (activation ≥ 2).

We next performed functional gene enrichment analyses using the Ingenuity Pathway Analysis (IPA) tool. The significantly regulated unique genes were merged for each stimulus. A core analysis was carried out and biological annotation enrichment was generated based on genes. The top 20 biological functions derived from calprotectin and IL27-regulated genes are shown in Figure 2. IL27-mediated gene expression showed that IL27 activated all of the presented top 20 biological functions, including, for example, inflammatory response and activation, stimulation, and migration of leukocytes (*z*-score ≥ 2). However, calprotectin only mediated the activation of 1 out of the 20 presented biological functions. This pathway analysis reveals that while IL27 appears to be involved in typical inflammatory functions, the activity of calprotectin is less obvious.

### 3.2. Calprotectin-Dependent Modulatory Effects on IL27-Mediated Gene Expression of* IL7*,* IL15*,* CXCL10*, and* CXCL11*

**Figure 3:**
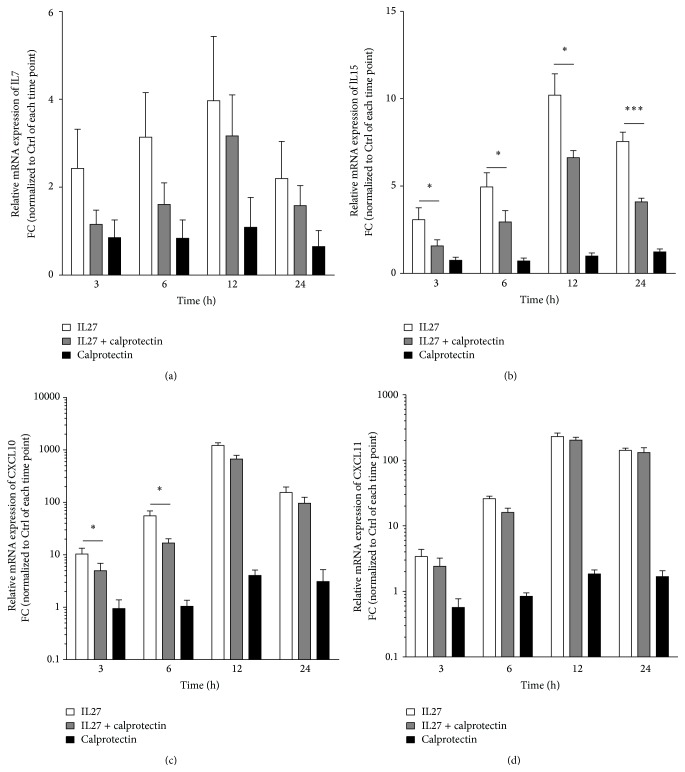
Effects of IL27, calprotectin, and IL27/calprotectin cotreatment on gene expression. Relative mRNA levels of IL7, IL15, CXCL10, and CXCL11 of IL27 (30 ng/mL) ± calprotectin (1 *μ*g/mL)-stimulated HUVECs for 3, 6, 12, and 24 h are represented as mean ± SEM (*n* = 6). Indicated *p* values are corresponding to significant differences between IL27 and IL27 + calprotectin: ^*∗*^ *p* < 0.05, ^*∗∗*^ *p* < 0.01, and ^*∗∗∗*^ *p* < 0.001.

To evaluate the possible role of calprotectin in the regulation of the expression of the IL27-dependent upregulated genes* IL7*,* IL15*,* CXCL10*, and* CXCL11*, HUVECs were treated with IL27 (30 ng/mL) ± calprotectin (1 *μ*g/mL) and the relative gene expression was determined by RT-qPCR for the different time points 3, 6, 12, and 24 h as shown in Figure 3. Calprotectin induced downregulation of IL27-mediated gene expression of* IL15* and* CXCL10* while no significant effect was observed neither on* IL7* nor on* CXCL11*. Interestingly, calprotectin decreased IL27-induced gene expression of* IL15* by half at all time points, whereas its downregulating effect on* CXCL10* was weaker and limited to the early time points 3 h and 6 h. These results point to a specific downregulatory role of calprotectin in the endothelial IL27-dependent signaling leading to gene expression of* IL15* and* CXCL10*.

**Figure 4:**
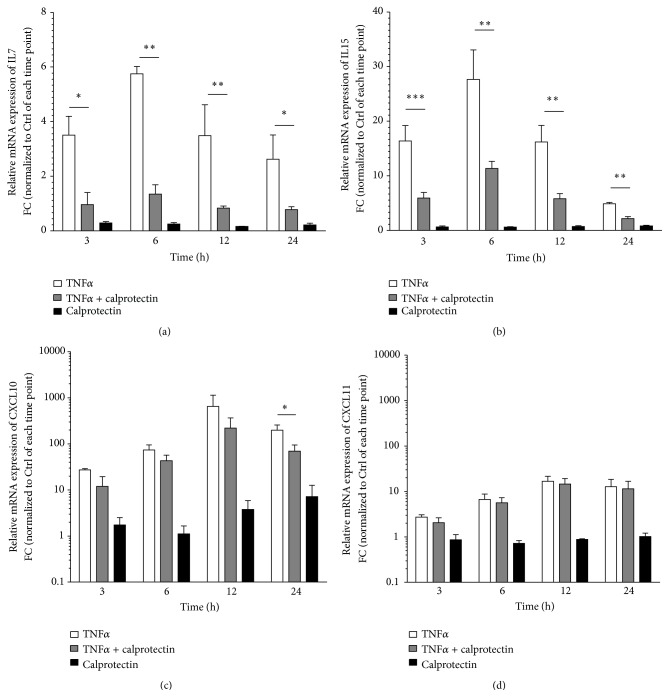
Effects of TNF*α*, calprotectin, and TNF*α*/calprotectin cotreatment on gene expression. Relative mRNA levels of IL7, IL15, CXCL10, and CXCL11 of TNF*α* (2 ng/mL) ± calprotectin (1 *μ*g/mL)-stimulated HUVECs for 3, 6, 12, and 24 h are represented as mean ± SEM (*n* = 3). Indicated *p* values are corresponding to significant differences between TNF*α* and TNF*α* + calprotectin: ^*∗*^ *p* < 0.05, ^*∗∗*^ *p* < 0.01, and ^*∗∗∗*^ *p* < 0.001.

In order to confirm the proinflammatory activity for IL27 and the calprotectin modulatory effects, the HUVECs were stimulated with TNF*α* (2 ng/mL) ± calprotectin (1 *μ*g/mL) for 3, 6, 12, and 24 h, and the gene expression of* IL7*,* IL15*,* CXCL10*, and* CXCL11* was analysed by RT-qPCR (Figure 4). We observed similar TNF*α*-mediated gene upregulation of* IL7*,* IL15*,* CXCL10*, and* CXCL11*. Furthermore, calprotectin induced downregulatory effects on TNF*α*-mediated gene expression of IL7, IL15, and CXCL10, hence validating our previous findings and emphasizing the* IL15*- and* CXCL10*-specific regulatory role of calprotectin.

### 3.3. Calprotectin-Dependent Modulatory Effects on IL27-Mediated Intracellular Protein Expression

**Figure 5:**
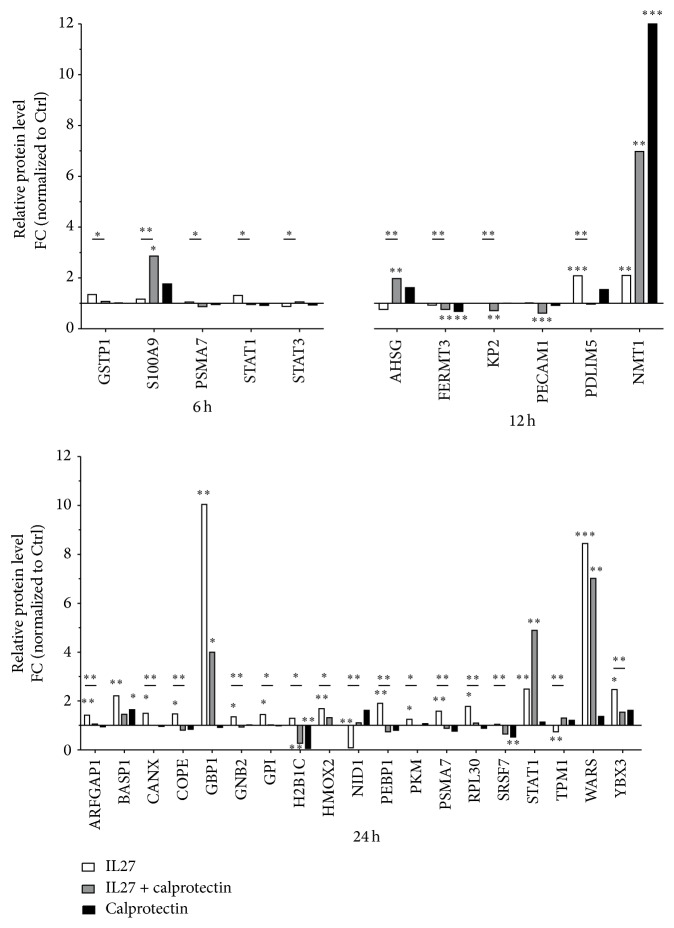
Label-free quantified proteins represented as relative protein levels. The calprotectin significant modulatory effects on mutual proteins in HUVECs stimulated with IL27 (30 ng/mL) ± calprotectin (1 *μ*g/mL) are shown for 6, 12, and 24 h (*n* = 9). Significance is indicated by *q*-value: ^*∗*^ *q* < 0.15, ^*∗∗*^ *q* < 0.10, and ^*∗∗∗*^ *q* < 0.01. Significance is indicated for each stimulation by stars and between IL27 and IL27 + calprotectin stimulation by stars with a line. AHSG, alpha-2-HS-glycoprotein; ARFGAP1, ADP-ribosylation factor GTPase-activating protein 1; BASP1, brain acid soluble protein 1; CANX, calnexin; COPE, coatomer subunit epsilon; FERMT3, fermitin family homolog 3; GBP1, interferon-induced guanylate binding protein 1; GNB2, guanine nucleotide-binding protein, subunit beta-2; GPI, glucose-6-phosphate isomerase; GSTP1, glutathione S-transferase P; H2B1C, histone H2B type 1; HMOX2, heme oxygenase 2; KP2, importin subunit alpha-1; NID1, nidogen-1; NMT1, glycylpeptide N-tetradecanoyl transferase 1; PDLIM5, PDZ and LIM domain protein 5; PEBP1, phosphatidylethanolamine-binding protein 1; PECAM1, platelet/endothelial cell adhesion molecule; PSMA7, proteasome subunit alpha type-7; RPL30, 60S ribosomal protein L30; S100A9, S100 calcium binding protein A9; SRSF7, serine/arginine-rich splicing factor 7; STAT, signal transducers and activators of transcription; TPM1, tropomyosin 1; WARS, tryptophan tRNA ligase, cytoplasmic; YBX3, Y-box-binding protein 3.

**Figure 6:**
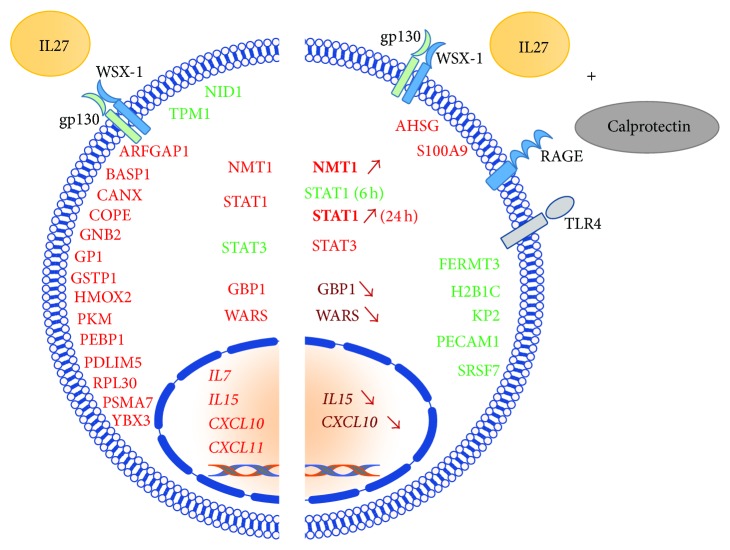
Qualitative representation of the calprotectin modulatory effects on IL27-mediated protein and gene expression in HUVECs. Colour code: red: upregulation, bold red (arrow up): increased upregulation compared to IL27 stimulation only, dark red (arrow down): decreased upregulation compared to IL27 stimulation only, and green: downregulation. IL27 receptor: gp130/WSX-1 and calprotectin receptors: RAGE (advanced glucated end product receptor) and TLR4 (Toll-like receptor 4).

To investigate the IL27-induced protein expression in HUVECs and to examine the potential regulatory role of calprotectin in this process, we used a label-free MS-based proteomic approach. HUVECs were stimulated with IL27 (30 ng/mL) ± calprotectin (1 *μ*g/mL) for 6, 12, and 24 h. In total, the number of unique proteins detected for each time point was 1061 at 6 h, 994 at 12 h, and 886 at 24 h (Table 1). As shown in Figure S4 by a representation of *q*-values over *p* values derived from proteome data, a significance cut-off threshold corresponding to *q*-value <0.15 could be chosen. The data indicated the following unique differentially regulated proteins detected for each time step: 11 at 6 h, 143 at 12 h, and 193 at 24 h (Table 1). Furthermore, a time-dependent increase for significant expressed proteins for IL27- and calprotectin-stimulated HUVECs was observed. Interestingly, the costimulation revealed already highest protein expression number at 12 h suggesting a calprotectin modulatory role in IL27-mediated protein expression (Table 1). As shown in Figure 5, calprotectin time-dependent modulatory effects on protein expression were observed on 28 unique proteins.  Figure 6 summarizes qualitatively the modulatory role of calprotectin in IL27-mediated protein expression. While calprotectin potentialized the IL27-dependent expression of NMT1 (12 h) and STAT1 (24 h) and upregulated STAT3 (6 h), it decreased the expression of GBP1 and WARS at 24 h and downregulated STAT1 at 6 h. Furthermore, calprotectin prevented the IL27-mediated downregulation of NID1 and TPM1, as well as the upregulation of 14 proteins: ARFGAP1, BASP1, CANX, COPE, GNB2, GP1, GSTP1, HMOX2, PKM, PEBP1, PDLIM5, RPL30, PSMA7, and YBX3. The results also showed that the costimulation of HUVECs with IL27 + calprotectin induced the downregulation of 5 proteins (FERMT3, H2B1C, KP2, PECAM1, and SRSF7) whereas 2 proteins (AHSG and S100A9) were upregulated. However, none of the proteins corresponding to the genes induced by IL27 and regulated by calprotectin were differentially expressed. These findings emphasize at the protein level the role played by calprotectin in the regulation of IL27-dependent protein expression.

### 3.4. Calprotectin-Dependent Modulatory Effects on IL27-Mediated STAT1/3 Signaling

**Figure 7:**
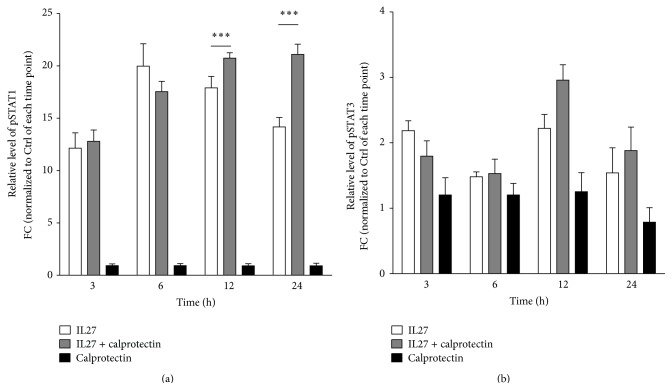
Effects of IL27, calprotectin, and IL27 + calprotectin cotreatment on STAT1/3 phosphorylation. Relative protein levels of Tyr701-phosphorylated pSTAT1 (a) and Tyr705-pSTAT3 (b) in IL27 (30 ng/mL) ± calprotectin (1 *μ*g/mL)-stimulated HUVECs for 3, 6, 12, and 24 h performed by western blot. Values are represented as mean ± SEM. Normalization was performed against tubulin. Presented statistical significances are between IL27 and IL27 + calprotectin and the indicated *p* values correspond to ^*∗∗∗*^ *p* < 0.001.

The signal transducers and activators of transcription (STAT) 1 and 3 are both known to be part of the signaling downstream cascade of IL27 and are activated through phosphorylation [49, 50]. Our proteomic results clearly indicate that calprotectin upregulated IL27-mediated STAT3 protein expression at 6 h whereas protein expression of STAT1 was downregulated at 6 h and upregulated at 24 h (Figure 5). We next analysed by western blotting the phosphorylation levels of STAT1 and STAT3 when HUVECs were stimulated with IL27 (30 ng/mL) ± calprotectin (1 *μ*g/mL) for 3, 6, 12, and 24 h (Figures 7 and S5). Interestingly, the IL27 + calprotectin cotreatment clearly potentialized the IL27-mediated STAT1 phosphorylation at 12 and 24 h but had no effect on the STAT3 phosphorylation over the time, suggesting that calprotectin might act through the regulation of the STAT1 activity to modulate the IL27-dependent proinflammatory signaling.

## 4. Discussion

The function of the cytokine IL27 in the inflammatory mechanism leading to immune cell transmigration through the endothelium is controversially discussed. In this study, we investigated the potential IL27-dependent induced inflammatory responses regarding endothelial cell gene and protein expression in the context of vascular inflammation. In addition, we propose that calprotectin may be involved in the regulation of this process.

### 4.1. The Modulatory Role of Calprotectin in IL27-Mediated Inflammation

Animal and human studies revealed contradictory observations for the investigation of the atherogenic role of the IL27. Studies in animal models have shown an atheroprotective role whereas human studies demonstrated an important proatherogenic role of IL27 in cardiovascular disease [30, 51]. Our findings demonstrate that exposure of HUVECs to different literature-based chosen IL27 concentrations results in differential gene expression of 19 out of 96 tested genes (Figure 1(a) and Table S1). Among these 19 genes, we observed 15 upregulated genes which are known to be involved in inflammation. Our pathway analysis revealed that the IL27-induced genes are highly involved in the activation of a variety of biological functions such as activation, stimulation, and migration of leukocytes (Figure 2). Since these functions are fundamental characteristics of the early steps of atherosclerosis development, we propose not only a proinflammatory but also a proatherogenic role for IL27, which is in agreement with the pathway analysis of King and coworkers [30].

In human studies, the two calprotectin subunits S100A8 and S100A9 were detected in atherosclerotic plaques and elevated in serum of patients suffering from peripheral artery disease [52, 53]. In our study, stimulating HUVECs with different calprotectin concentrations revealed differential gene expression of 16 out of 96 tested genes (Figure 1(b) and Table S1). However, calprotectin-induced gene expression indicated poor regulatory effects in terms of expression level (only 2 genes with FC > 1.5). Our observations are in accordance with a study of Viemann and collaborators in which human microvascular ECs were stimulated with 200 *μ*g/mL calprotectin for 6 h: although the calprotectin concentration used was elevated, only 16 genes with a FC > 1.7 were induced and proposed to be involved in promoting platelet aggregation, inflammation, and endothelial permeability [54].

In order to enlighten the role of calprotectin in our experimental setup, our pathway analysis indicated that the calprotectin-regulated genes were not implicated in the typical activation and migration of leukocytes but solely involved in the delayed hypersensitive reaction (Figure 2). Based on this pathway analysis, we hypothesized a possible calprotectin modulatory activity in inflammatory response of HUVECs. In order to clarify the inflammatory role of calprotectin, we investigated their potential synergistic, additive, or antagonistic effects on transcriptome and proteome levels. We first analysed the impact of calprotectin on HUVEC gene expression level by focussing on the 4 most upregulated genes upon IL27 stimulation (*IL7*,* IL15*,* CXCL10*, and* CXCL11*) (Figure 3).

We observed clear calprotectin modulatory effects on IL27-mediated* IL15* and* CXCL10* gene expression (Figures 3(b) and 3(c)). Interestingly, the stimulation with calprotectin reduced the IL27-mediated gene expressions of* IL15* over the entire studied time range and of* CXCL10* at the early time points (Figures 3(b) and 3(c)). Based on our observations, we propose an anti-inflammatory role of calprotectin in the IL27-mediated* IL15* and* CXCL10* gene expression. But the cytokine IL27 is known to have pleiotropic functions with pro- and anti-inflammatory capacities [20], which could lead to misinterpretation of the anti-inflammatory function proposed for calprotectin. Thus, we also stimulated HUVECs with the potent proinflammatory TNF*α* in the presence of calprotectin in the same conditions. Interestingly, similar calprotectin reducing effects were observed on TNF*α*-mediated gene expression (Figure 4), which highly supports our hypothesis about the anti-inflammatory capacity of calprotectin on IL27-stimulated HUVECs. Analysing only the genome is not sufficient to completely understand a phenotype or a disease development process. Therefore we further analysed the intracellular proteome of whole cell lysates of IL27 ± calprotectin-stimulated HUVECs by a shot-gun label-free LC-MS/MS approach to gain greater insight into our proposed anti-inflammatory capacity of calprotectin in the context of endothelial inflammation. The label-free quantification revealed a number of identified and quantified proteins similar to the one of Gautier et al. [55], who identified 725 unique proteins in untreated HUVEC samples. Another shot-gun proteomic approach combined with label-free quantification was also successfully applied to HUVECs treated with a clinical phase III candidate [56]. In order to further proceed with our data, we used the positive false discovery rate also known as *q*-value, which controls the false discoveries and corrects for multiple testing to calculate the significance [57]. Indeed *q*-values were shown to provide a more direct way of interpreting significance than the *p* value in the context of quantitative proteomics [45]. In our study, the direct comparison between *p* values and *q*-values using the well accepted significance threshold for *p* value <0.05 revealed a *q*-value threshold of maximum 0.175 (Figure S4). Based on this result, we chose a significance threshold of *q*-value <0.15, meaning that less than 15% of our significant regulated proteins are false discoveries. This finally revealed the numbers shown in Table 1 for significant regulated proteins.

Our proteomic approach revealed that calprotectin modulated 28 unique proteins (Figure 5). Unfortunately, the sample complexity of our whole HUVEC cell lysates did not allow the determination of low abundant proteins such as cytokines. Thus, the calprotectin inhibitory effect on IL27-mediated gene expression of* IL15* and* CXCL10* could not be examined on protein level by shot-gun LC-MS/MS. However, higher protein levels of CXCL10 as well as CXCL11 were detected in human and carotid atherosclerotic tissues whereas none of them were detected in normal vessel walls, highlighting that both proteins are playing an important role in the development of atherosclerosis [58].

**Figure 8:**
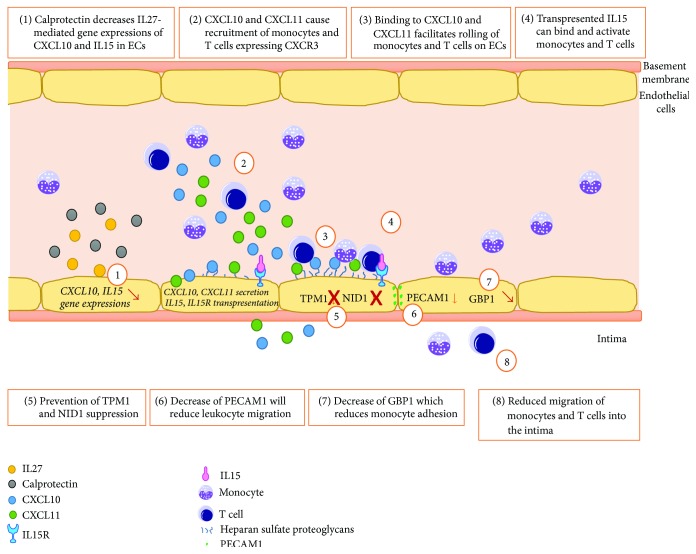
Hypothetical model of the calprotectin modulatory effects on IL27-mediated gene and protein expression based on our experimental results and literature data. GBP1, guanylate binding protein 1; NID1, nidogen-1; PECAM1, platelet endothelial cell adhesion molecule; TPM1, tropomyosin 1.

Regarding our results, we therefore propose a model of a calprotectin reducing effect on IL27-mediated inflammation of the vascular endothelium in the context of atherosclerosis.  Figure 8 describes the possible mechanism of IL27-induced vascular inflammation and the potential atheroprotective role of calprotectin in this context. IL27 induced the secretion of the chemokines CXCL10 and CXCL11 which are known to attract monocytes as well as T cells. In more detail, CXCL10 and CXCL11 mainly recruit CD4 or CD8 T cells, which also represent the main T cell subsets found in atherosclerotic plaques [58–60]. CXCL10 and CXCL11 can bind via their cognate receptor CXCR3 which is highly expressed on monocytes, T cells, and NK cells [61]. In the atherosclerotic context, endothelial cells do not express CXCR3 but use a very defined system [62]. CXC chemokines such as CXCL10 and CXCL11 can also bind, for example, to heparan sulfate proteoglycans, which are present at the cell surface of endothelial cells. This binding of CXCL10 and CXCL11 can facilitate the rolling of monocytes and T cells on the vascular endothelium [62, 63]. Furthermore, it can facilitate the adhesion to other proteins such as the transpresented IL15 at the surface of endothelial cells. T cells and monocytes express the counterparts IL2R*α* and IL2R for IL15R to form the tridimeric IL15 receptor [64]. Independent studies have shown the transendothelial migration of T cells and monocytes through IL15 expression on endothelial cells [64, 65].

Our proteome analysis revealed an IL27-induced regulation of a variety of proteins which are involved in the inflammatory state of the endothelium as well as the development of atherosclerosis. For example, the extracellular matrix glycoprotein nidogen-1 (NID1) was highly downregulated by IL27. NID1 belongs to the basement membrane and is an important linker between the extracellular matrix components collagen and laminin [66]. It could be shown that suppression of NID1 can influence the cell morphology from a flat to a round shape by losing contact to the underlying basement membrane. Thus, an important functional implication of NID1 was suggested in the blood-vessel tissue barrier [67]. Furthermore, NID1 may be involved in the defense against infiltration of cancer cells [68]. Another highly downregulated protein by IL27 was tropomyosin 1 (TPM1), which is known as a tumour suppressor. Suppression of TPM1 can lead to the destabilization of the cytoskeleton [69]. It was described that the initiation and growth of atheroma can occur due to the loss of integrity of an intact endothelial monolayer [70]. In the context of atherosclerosis, a potential downregulator of TPM1 could be the small noncoding microRNA miR21 [71], as miR21 was the highest upregulated miRNA in a study of human atherosclerotic plaques [72]. In our study, IL27 induced the upregulation of GBP1 which was also observed in IFN*γ*-treated HUVECs [73], leading to enhanced adhesion of monocytes. Similar results of increased adherence of monocytes were demonstrated in stimulated Human Dermal Microvascular Endothelial Cell (HDMEC) and Human Coronary Artery Endothelial Cells (HCAEC) [74].

Based on our data and our hypothetical model, IL27-induced endothelial inflammation is involved in the recruitment, adhesion, and infiltration of monocytes and of T cells. Thus, we propose an important proatherogenic role for IL27 in the early steps of atherosclerosis. Furthermore, we observed IL27-induced upregulation of PEBP1 which is consistent with elevated PEBP1 levels found in atherosclerotic apoE^−/−^ mice [75]. We also propose an atheroprotective role for calprotectin, as illustrated in Figure 8. Stimulation with calprotectin prevented the IL27-mediated upregulation of NID1 and TPM1, suggesting that no impairment of the underlying basement membrane is occurring. In addition, calprotectin reduced IL27-mediated upregulation of the GBP1 which may lead to reduced monocyte adhesion to the endothelium. Interestingly, calprotectin downregulated the protein expression of PECAM1, leading to the assumption of reduced transendothelial migration: PECAM1 is indeed known as an important contributor for transendothelial migration of leukocytes into the vessel wall [76].

Comparing the stimulations by IL27 and by IL27 + calprotectin by IPA pathway analysis (Table S2), we identified that, regarding the costimulation, calprotectin was responsible for the inhibition of mTOR as well as of EIF2 signaling pathways. Different groups have observed in animal and human models that inhibition of mTOR can result in antiatherosclerotic effects causing prevention or delay of the pathogenesis of atherosclerosis [77, 78]. Based on our results, we propose an atheroprotective role for calprotectin through its IL27-dependent effect on the transendothelial migration of leukocytes into the vessel wall.

### 4.2. Hypotheses Regarding the Immunoprotective Role of Calprotectin

It has been demonstrated* in vivo* that calprotectin reduced LPS-mediated inflammation by binding directly to LPS but also to other cytokines such as IL1*β*, IL6, and TNF*α* [37]. Another* in vivo* animal study revealed a reduction of severe infiltration of inflammatory cells after calprotectin administration and suggested a scavenger activity for calprotectin [38]. Regarding our experimental setup, based on the knowledge that calprotectin can bind to TNF*α*, and since we showed that calprotectin similarly reduced both TNF*α*- and IL27-mediated gene expression, we also suggest a potential protein-protein interaction between calprotectin and IL27. This scavenger function does not directly imply a prevention of receptor binding and subsequent activation of the signaling pathway, as it depends on the availability of free binding sites of IL27. Whether calprotectin can prevent the binding of IL27 to its receptor requires more investigations. Besides, it is also well documented that IL27 receptor activation occurs through Janus kinases and STATs. Interestingly, a study identified that excretory/secretory (ES) products from a parasite reduced IFN*γ*-mediated* CXCL10* gene expression without any suppression of the phosphorylation-dependent activation of the upstream regulator STAT1 [79]. However, no decrease of* CXCL9* mRNA level was observed, which may be explained by the presence of different transactivating cofactors required for gene transcription. Whether this regulation process could be extrapolated to the observed different modulatory effects of calprotectin on IL27-mediated STAT-dependent* CXCL10*/*CXCL11* gene expression would require further investigations.

## 5. Conclusion

In conclusion, our findings clearly demonstrate that the cytokine IL27 plays an important role as a proinflammatory mediator by regulating the expression of endothelial genes and proteins, which may be mainly involved in the early stages of the atherosclerosis mechanism. More importantly, we show for the first time that calprotectin acts as a modulator of this process. Our main findings are the identification of a role of calprotectin in the regulation of IL27-mediated gene expression (*IL15*,* CXCL10*), protein expression (TPM1, NID1, PECAM1, and GBP1), and signaling activation (STAT1). Based on these observations, we suggest that, in the context of the IL27-induced vascular inflammation, calprotectin might be a novel attractive candidate as a regulator of monocyte recruitment to early atherosclerotic lesions, hence preventing the progression of inflammation and atherosclerosis.

## Supplementary Material

Figures S1, S2, S3, and S4 are related to the normalization and analysis of the proteomic data. Figure S5 shows one western blot used to obtain Figure 7 about STATs phosphorylation state. Table S1 shows the IL27/calprotectin contrast analysis using the multi-gene array TaqMan assay. Table S2 corresponds to the IPA analysis of IL27 versus IL27+calprotectin cell stimulation.

## Figures and Tables

**Table 1:** Number of unique identified proteins without and with *q*-value cut-off of *q* < 0.15 of IL27 (30 ng/mL) ± calprotectin (1 *μ*g/mL)-stimulated HUVECs for 6, 12, and 24 h.

	Time point		
	6 h	12 h	24 h
Unique proteins			
All identified	1061	994	886
*q*-value < 0.15	11	143	193

Condition (*q*-value < 0.15)			
IL27	0	55	132
IL27 + calprotectin	10	96	20
Calprotectin	1	17	66
