# Supplementary material for: Role of Calprotectin as a Modulator of the IL27-Mediated Proinflammatory Effect on Endothelial Cells
Source: Mediators Inflamm. 2015 Nov 17;2015:737310. doi: 10.1155/2015/737310 (PMC4664814; doi:10.1155/2015/737310)
Supplement: Supplementary file 1 — Figures S1, S2, S3, and S4 are related to the normalization and analysis of the proteomic data. Figure S5 shows one western blot used to obtain Figure 7 about STATs phosphorylation state. Table S1 shows the IL27/calprotectin contrast analysis using the multi-gene array TaqMan assay. Table S2 corresponds to the IPA analysis of IL27 versus IL27+calprotectin cell stimulation. [file 737310.f1.pdf]

Supplementary material

1. Transcriptome data

Table S1: Gene regulatory response of HUVECs stimulated with IL27 (10, 30, and 100 ng/mL) and calprotectin (1, 5, and 10 μg/mL) after 12 h. Multi-gene array TaqMan Immune human panel was used, genes with no indicated FC and p-values were not detected. (FC: Fold Change, p-val: p-value based on unpaired t.test).

	IL27									Calp								
	10			30			100			1			5			10		
Genes	FC	p-val		FC	p-val		FC	p-val		FC	p-val		FC	p-val		FC	p-val	
18S	1.31	0.14		1.04	0.85		1.16	0.58		0.95	0.70		0.91	0.50		1.05	0.74	
ACE	1.11	0.29		1.00	0.97		0.80	0.06		1.15	0.03	*	1.14	0.04	*	1.10	0.00	**
ACTB																		
AGTR1																		
AGTR2																		
BAX	0.98	0.80		0.93	0.24		1.02	0.71		0.83	0.05		0.93	0.36		0.90	0.12	
BCL2	0.79	0.48		0.95	0.88		0.77	0.46		1.02	0.71		1.04	0.63		1.13	0.03	*
BCL2L1	0.90	0.17		1.04	0.65		1.17	0.03	*	0.94	0.17		1.11	0.15		1.30	0.00	**
C3	1.68	0.02	*	1.71	0.02	*	1.17	0.50		0.95	0.71		1.15	0.35		0.84	0.32	
CCL19																		
CCL2	1.17	0.03	*	1.41	0.02	*	1.29	0.11		1.04	0.35		0.90	0.05	*	0.97	0.57	
CCL3																		
CCL5	1.03	0.87		1.23	0.23		1.18	0.41		4.83	0.02	*	0.83	0.44		1.52	0.52	
CCR2																		
CCR4	0.84	0.67		0.80	0.70		0.82	0.69		1.14	0.46		1.11	0.60		1.13	0.43	
CCR5																		
CCR7																		
CD19																		
CD28	0.94	0.86		1.52	0.34		0.62	0.29		0.90	0.84		1.14	0.70		1.11	0.73	
CD34	0.99	0.90		0.85	0.03	*	0.81	0.02	*	0.99	0.85		0.96	0.60		0.97	0.41	
CD38	2.17	0.08		2.52	0.00	***	2.29	0.08		1.20	0.07		1.09	0.50		1.09	0.33	
CD3E																		
CD40	1.18	0.21		1.10	0.57		1.12	0.43		1.60	0.44		1.77	0.47		1.16	0.79	
CD40LG																		
CD4	0.82	0.47		0.95	0.89		0.91	0.80										
CD68	0.87	0.20		0.81	0.18		0.79	0.10		0.91	0.13		0.87	0.03	*	1.00	0.97	
CD80																		
CD86																		
CD8A																		

Continued on next page

Continued from previous page

	IL27									Calp								
	10			30			100			1			5			10		
**Genes**	**FC**	**p-val**		**FC**	**p-val**		**FC**	**p-val**		**FC**	**p-val**		**FC**	**p-val**		**FC**	**p-val**	
COL4A5	1.11	0.38		1.05	0.68		1.00	0.98		1.11	0.25		1.02	0.80		1.07	0.42	
CSF1	1.55	0.01	**	1.78	0.00	**	1.64	0.04	*	1.00	0.93		0.98	0.37		1.11	0.02	*
CSF2	0.96	0.93		1.07	0.89		2.21	0.05	*	1.10	0.74		0.82	0.44		1.27	0.53	
CSF3	0.64	0.27		2.26	0.08		0.91	0.84		1.13	0.48		1.35	0.23		1.45	0.09	
CTLA4																		
CXCL10	51.26	0.00	***	140.13	0.00	***	146.94	0.00	***	1.08	0.52		0.98	0.90		0.86	0.27	
CXCL11	40.94	0.00	***	77.88	0.00	***	85.51	0.00	***	1.09	0.22		0.97	0.73		1.14	0.08	
CXCR3																		
CYP1A2																		
CYP7A1	0.95	0.83		0.51	0.28		0.93	0.93		0.52	0.01	**	0.64	0.38		0.71	0.23	
ECE1	0.91	0.51		0.98	0.89		1.00	0.99		1.12	0.22		0.97	0.67		1.01	0.94	
EDN1	0.98	0.80		0.90	0.22		0.99	0.92		0.93	0.32		0.88	0.07		0.96	0.39	
FAS	1.59	0.03	*	1.68	0.02	*	1.54	0.03	*	1.05	0.35		1.06	0.39		0.95	0.29	
FASLG																		
FN1	1.07	0.27		1.01	0.92		0.95	0.41		1.02	0.78		1.01	0.94		0.93	0.23	
GAPDH																		
GNLY																		
GUSB																		
GZMB																		
HLA																		
HLA																		
HMOX1	0.89	0.42		0.89	0.43		1.03	0.85		0.98	0.78		0.95	0.57		1.08	0.39	
ICAM1	1.28	0.03	*	1.65	0.03	*	2.08	0.00	***	1.05	0.51		0.94	0.48		1.12	0.19	
ICOS																		
IFNG																		
IKBKB	0.86	0.26		0.89	0.34		0.70	0.02	*	1.00	0.93		0.88	0.05		0.92	0.14	
IL10																		
IL12A	1.98	0.00	***	1.92	0.05	*	1.95	0.04	*	1.14	0.21		0.84	0.12		1.03	0.80	

Continued on next page

Continued from previous page

	**IL27**									**Calp**								
	**10**			**30**			**100**			**1**			**5**			**10**		
**Genes**	**FC**	**p-val**		**FC**	**p-val**		**FC**	**p-val**		**FC**	**p-val**		**FC**	**p-val**		**FC**	**p-val**	
IL12B																		
IL13																		
IL15	6.53	0.00	***	6.22	0.00	**	5.75	0.00	**	0.87	0.48		0.80	0.23		0.83	0.43	
IL17																		
IL18	1.45	0.32		0.88	0.71		0.84	0.65		0.97	0.91		1.19	0.47		1.02	0.94	
IL1A	0.83	0.37		0.84	0.53		0.79	0.27		1.09	0.38		0.92	0.55		1.01	0.96	
IL1B	0.92	0.58		1.47	0.23		2.26	0.01	**	0.90	0.46		0.83	0.20		1.06	0.52	
IL2																		
IL2RA																		
IL3																		
IL4																		
IL5																		
IL6	1.53	0.08		2.23	0.01	**	2.19	0.02	*	1.13	0.46		1.00	1.00		1.12	0.36	
IL7	7.42	0.00	***	6.80	0.00	***	4.40	0.03	*	2.19	0.01	**	1.42	0.37		1.41	0.29	
IL8	0.73	0.27		0.76	0.44		0.92	0.72		1.06	0.64		0.90	0.48		1.06	0.64	
IL9																		
LRP2																		
LTA	1.20	0.37		1.09	0.58		0.85	0.02	*	1.13	0.29		0.66	0.07		0.88	0.59	
MYH6																		
NFKB2	0.72	0.02	*	0.85	0.28		0.75	0.03	*	0.95	0.48		0.89	0.16		1.02	0.79	
NOS2A	0.67	0.07		1.37	0.11		1.16	0.36		1.03	0.94		0.80	0.21		1.52	0.05	*
PGK1	0.92	0.56		1.00	0.98		0.94	0.69		0.84	0.03	*	0.89	0.20		0.78	0.01	*
PRF1																		
PTGS2	1.13	0.64		0.99	0.97		1.26	0.48		1.07	0.01	*	0.92	0.01	**	1.14	0.00	**
PTPRC																		
REN																		
RPL3L																		
SELE	0.53	0.22		0.89	0.79		0.62	0.38		1.10	0.31		0.78	0.04	*	0.80	0.05	

Continued on next page

Continued from previous page

	IL27						Calp								
	10		30		100		1			5			10		
Genes	FC	p-val	FC	p-val	FC	p-val	FC	p-val		FC	p-val		FC	p-val	
SELP	1.10	0.12	0.99	0.81	1.02	0.64	1.26	0.01	**	1.17	0.02	*	1.29	0.00	**
SKI	1.04	0.56	1.00	0.98	0.96	0.62	1.12	0.22		1.03	0.78		1.10	0.27	
SMAD3	0.93	0.70	1.10	0.64	0.97	0.89	1.08	0.23		1.09	0.39		1.25	0.01	**
SMAD7	1.09	0.51	0.98	0.85	0.98	0.86	1.01	0.79		1.05	0.44		0.99	0.76	
STAT3	1.16	0.22	1.19	0.16	1.13	0.45	0.96	0.23		0.89	0.03	*	1.00	0.97	
TBX21															
TFRC	1.04	0.69	1.03	0.78	0.94	0.55	1.05	0.34		0.99	0.90		0.93	0.19	
TGFB1	1.08	0.14	1.02	0.58	1.05	0.41	0.91	0.37		0.86	0.05		0.97	0.58	
TNF															
TNFRSF18	0.63	0.55	2.82	0.29	6.55	0.10	0.60	0.41		1.49	0.74		0.63	0.48	
VEGF	0.95	0.71	0.89	0.39	0.91	0.60	0.96	0.64		0.89	0.11		0.94	0.34	

2. Analysis and normalization of proteome data

Assessment of protein abundance densities was performed to compare the individual replicates of each stimulatory condition and time point (Figure S1). Some replicates showed differences in protein abundance densities compared to the majority of overlapping protein abundance densities (Figure S1 A-C). Therefore, a further comparison by calculation of the peak sums for every sample was performed. Figure S2 shows the peak sums of protein abundances for every replicate per condition and time point. A median of all peak sums per time point was calculated and is shown as black lines. Application of a threshold standard deviation (sd) of 1.25 caused the elimination of replicates representing a different protein abundance density compared to the majority. Using this approach, we excluded 2 replicates for the 6 h time point, 6 replicates for the 12 h time point, and 7 replicates for the 24 time point. Analysis of the data distribution revealed to be variable and asymmetric by representing the sd over mean of protein abundances per time. Therefore, a variance stabilization and normalization (VSN) was applied and sd over mean of transformed protein abundances remained stable (Figure S3).

Significance was calculated based on Storey's positive false discovery rate also known as q.value. A comparative study between q.value and p-value revealed that a p-value ≤ 0.05 result in a q.value of 0.175 (Figure S4).

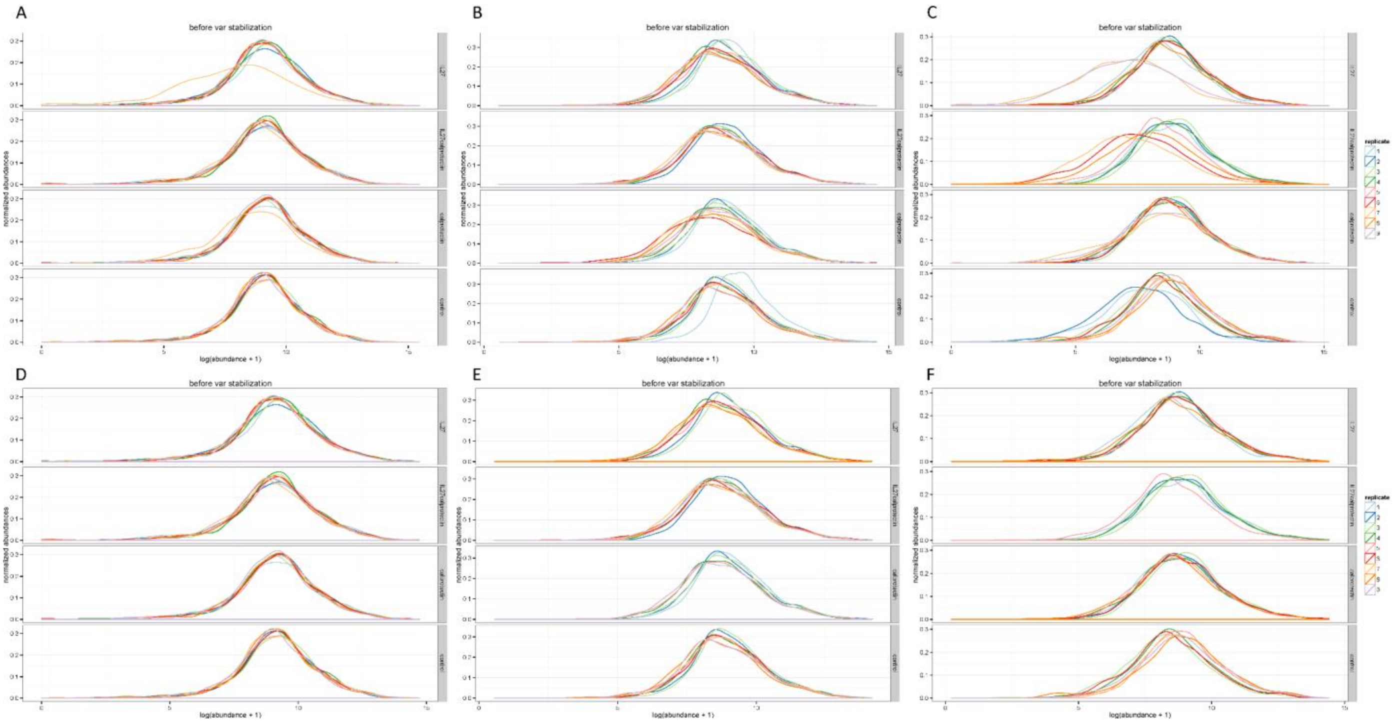

Figure S1: Protein abundances presented as densities of time points 6, 12, and 24 h. A, B, and C: before elimination of replicates at 6, 12, and 24 h respectively. D, E, and F: after elimination of replicates.

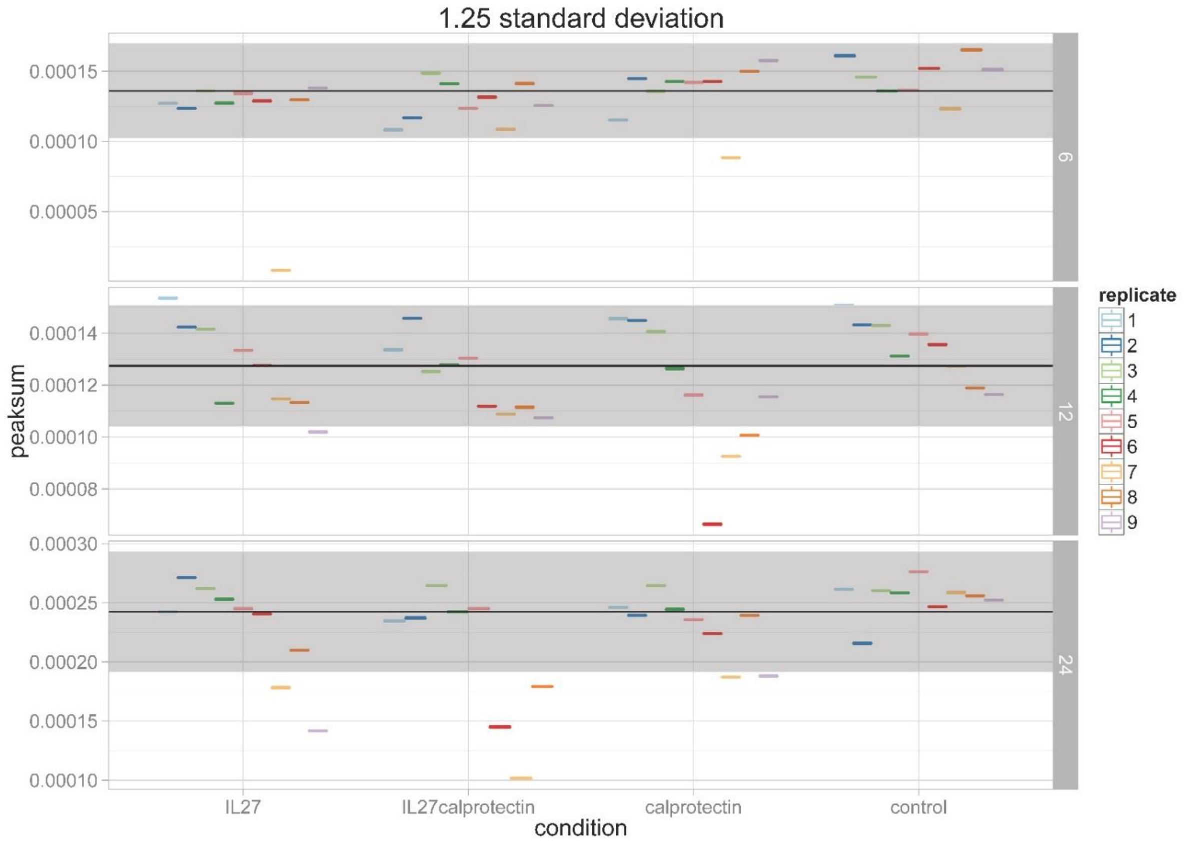

Figure S2: Elimination of replicates based on peak sums of protein abundances and application of a threshold standard deviation (sd) of 1.25 (indicated in grey). Time points 6, 12, and 24 h are presented separately and the black lines indicate the median of all peak sums for each time point. Peak sum per replicates is represented by a colour code.

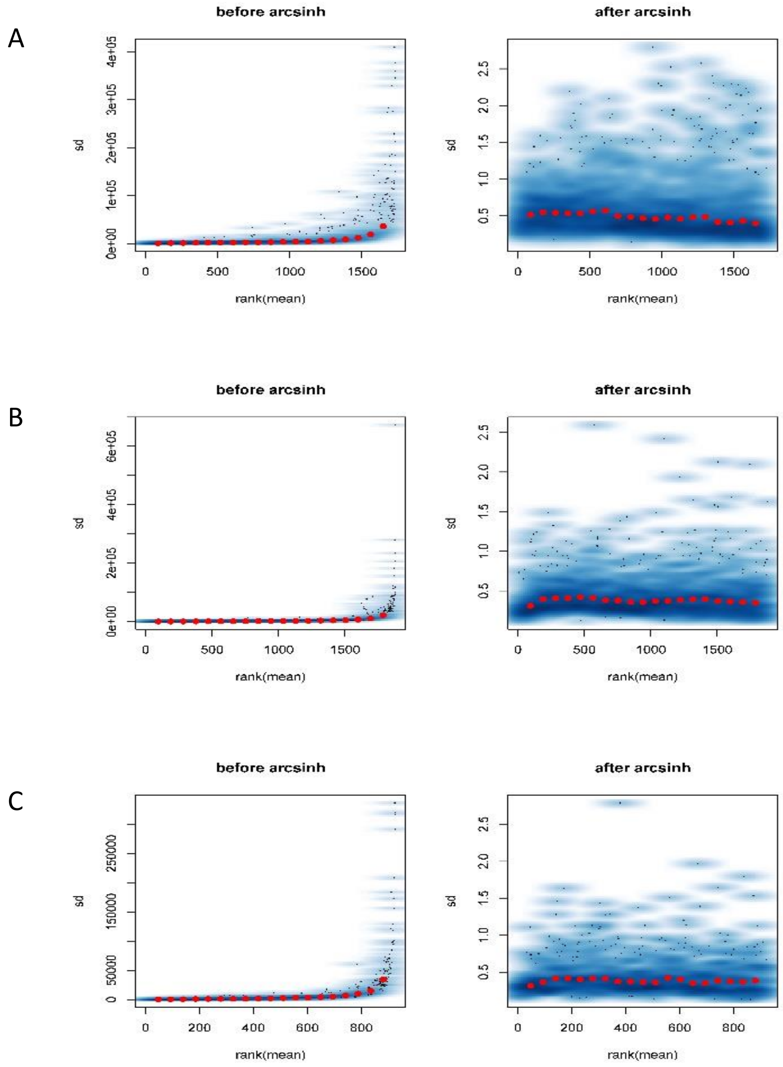

Figure S3: Standard deviation over mean before and after applying arcsinh function (variance stabilization and normalization (VSN)) of 6, 12, and 24 h time points.

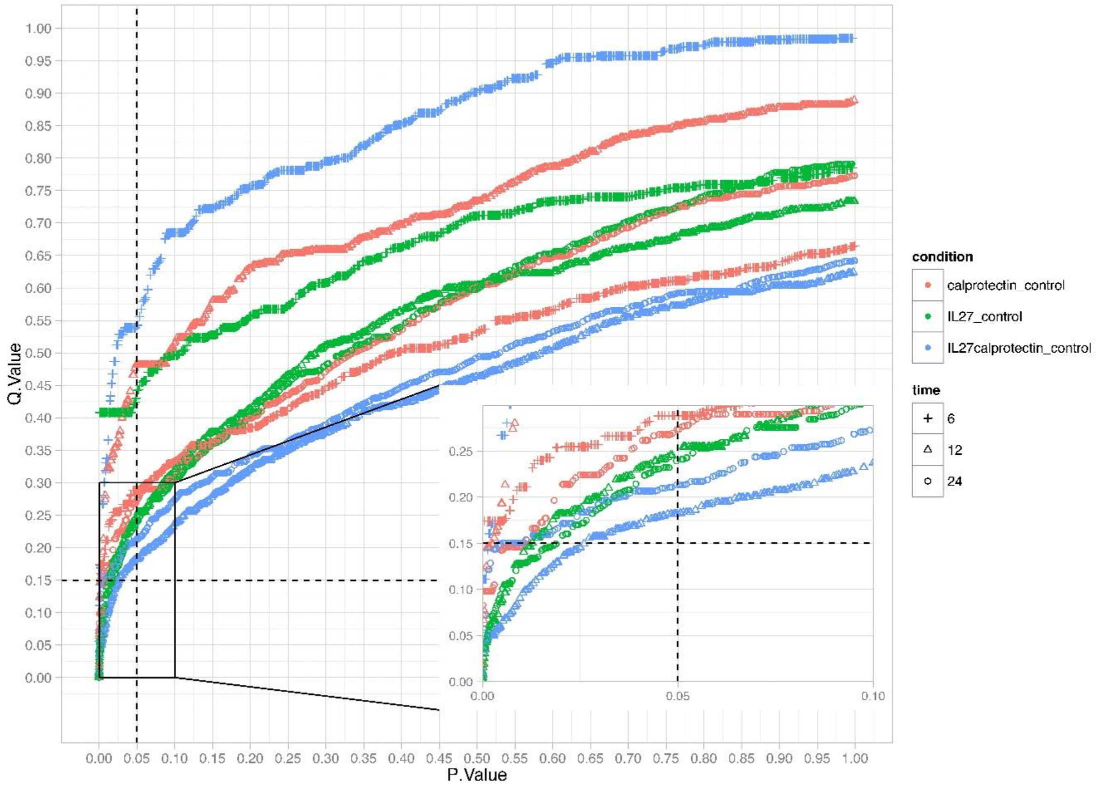

Figure S4: Comparison of p-values and q.values to identify a significance threshold for the q.values. A q.value of 0.15 was chosen as significance threshold.

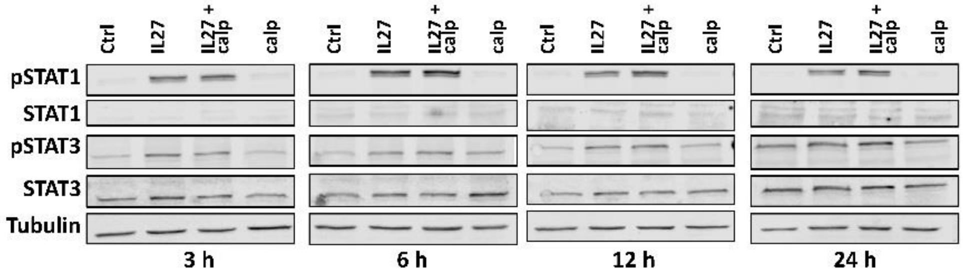

Figure S5: Western blot of LI-COR detected pSTAT1, STAT1, pSTAT3, STAT3, and tubulin in HUVECs stimulated with IL27 (30 ng/mL) ± calprotectin (1 μg/mL) for 3, 6, 12, and 24 h**.**

Table S2: Core analysis performed by IPA showing the top canonical pathways after stimulation with IL27 (30 ng/mL) ± calprotectin (1 μg/mL) for each stimulus vs Ctrl and also the comparison between IL27 and IL27 + calprotectin. Abbreviation: not a number (NAN).

**IL27**			
**Name**	**p-value**	**z-score**	**Proteins**
Signalling by Rho Family GTPases	$6.30 \cdot 10^{-7}$	0	12
Remodelling of Epithelial Adherens Junction	$1.44 \cdot 10^{-6}$	0	7
Protein Ubiquitination Pathway	$1.68 \cdot 10^{-6}$	NAN	12
RhoGDI Signalling	$2.40 \cdot 10^{-6}$	0.707	10
Actin Cytoskeleton Signalling	$1.54 \cdot 10^{-6}$	NAN	10

**IL27+calprotectin**			
**Name**	**p-value**	**z-score**	**Proteins**
Protein Ubiquitination Pathway	$2.77 \cdot 10^{-7}$	NAN	11
EIF2 Signalling	$7.29 \cdot 10^{-4}$	NAN	6
mTOR Signalling	$8.58 \cdot 10^{-4}$	NAN	6
Antigen Presentation Pathway	$1.21 \cdot 10^{-3}$	NAN	3
Regulation of eIF4 and p70S6K Signalling	$1.68 \cdot 10^{-3}$	NAN	5

**calprotectin**			
**Name**	**p-value**	**z-score**	**Proteins**
EIF2 Signalling	$1.30 \cdot 10^{-10}$	-2	11
mTOR Signalling	$5.19 \cdot 10^{-8}$	NAN	9
Regulation of EIF4 and p70S6K Signalling	$1.02 \cdot 10^{-7}$	NAN	8
Germ Cell-Sertoli Cell Junction Signalling	$3.90 \cdot 10^{-4}$	NAN	5
14-3-3-mediated Signalling	$1.09 \cdot 10^{-3}$	NAN	4

**IL27 vs IL27+calprotectin**			
**Name**	**p-value**	**z-score**	**Proteins**
EIF2 Signalling	$9.26 \cdot 10^{-17}$	1.155	25
Regulation of EIF4 and p70S6K Signalling	$1.20 \cdot 10^{-11}$	1.342	18
Protein Ubiquitination Pathway	$2.53 \cdot 10^{-9}$	NAN	20
mTOR Signalling	$6.16 \cdot 10^{-9}$	2.449	17
Remodelling of Epithelial Adherens Junction	$7.70 \cdot 10^{-7}$	NAN	9
